# Integration of Dynamical Network Biomarkers, Control Theory and *Drosophila* Model Identifies Vasa/DDX4 as the Potential Therapeutic Targets for Metabolic Syndrome

**DOI:** 10.3390/cells14060415

**Published:** 2025-03-12

**Authors:** Kazutaka Akagi, Ying-Jie Jin, Keiichi Koizumi, Makito Oku, Kaisei Ito, Xun Shen, Jun-ichi Imura, Kazuyuki Aihara, Shigeru Saito

**Affiliations:** 1 Division of Presymptomatic Disease, Institute of Natural Medicine, University of Toyama, Toyama 930-0194, Japan; kakagi@inm.u-toyama.ac.jp; 2 Research Center for Pre-Disease Science, University of Toyama, Toyama 930-8555, Japan; oku@cts.u-toyama.ac.jp (M.O.); s30saito@med.u-toyama.ac.jp (S.S.); 3 Graduate School of Pharma-Medical Sciences, University of Toyama, Toyama 930-0194, Japan; homura19961014@gmail.com; 4 Department of Pharmaceutical Sciences, School of Pharmacy and Pharmaceutical Sciences, University of Toyama, Toyama 930-0194, Japan; s2160304@ems.u-toyama.ac.jp; 5 Graduate School of Bio-Applications and Systems Engineering, Tokyo University of Agriculture and Technology, Tokyo 184-8588, Japan; shen@go.tuat.ac.jp; 6 Department of Systems and Control Engineering, School of Engineering, Institute of Science Tokyo, Tokyo 152-8552, Japan; imura.j.6486@m.isct.ac.jp; 7 International Research Center for Neurointelligence (WPI-IRCN), The University of Tokyo, Tokyo 113-0033, Japan; kaihara@g.ecc.u-tokyo.ac.jp

**Keywords:** dynamical network biomarkers theory, DNB intervention analysis, metabolic syndrome, *Drosophila melanogaster*

## Abstract

Metabolic syndrome (MetS) is a subclinical disease, resulting in increased risk of type 2 diabetes (T2D), cardiovascular diseases, cancer, and mortality. Dynamical network biomarkers (DNB) theory has been developed to provide early-warning signals of the disease state during a preclinical stage. To improve the efficiency of DNB analysis for the target genes discovery, the DNB intervention analysis based on the control theory has been proposed. However, its biological validation in a specific disease such as MetS remains unexplored. Herein, we identified eight candidate genes from adipose tissue of MetS model mice at the preclinical stage by the DNB intervention analysis. Using *Drosophila*, we conducted RNAi-mediated knockdown screening of these candidate genes and identified *vasa* (also known as *DDX4*), encoding a DEAD-box RNA helicase, as a fat metabolism-associated gene. Fat body-specific knockdown of *vasa* abrogated high-fat diet (HFD)-induced enhancement of starvation resistance through up-regulation of triglyceride lipase. We also confirmed that DDX4 expressing adipocytes are increased in HFD-fed mice and high BMI patients using the public datasets. These results prove the potential of the DNB intervention analysis to search the therapeutic targets for diseases at the preclinical stage.

## 1. Introduction

Metabolic syndrome (MetS) is a cluster of conditions including abdominal obesity, hypertension, hyperglycemia, and dyslipidemia. Clinically, persons who have two or more of these conditions are diagnosed with MetS. Consideration should be given to the fact that MetS patients and even non-MetS persons having one of these conditions potentially risk serious complications such as type 2 diabetes (T2D), cardiovascular disease, and associated morbidities [1]. Accordingly, MetS is considered as a preclinical (pre-disease) stage of these diseases. It is possible that biological changes have already occurred in the cellular level at a very early stage; however, detecting these early signs of the disease at a preclinical stage is still challenging.

The research models for studying MetS have been established using rodent models as well as non-mammalian model organisms such as *Drosophila melanogaster*, *C. elegans*, and zebrafish [2]. *Drosophila* shares the conserved endocrine mechanisms with mammals that regulate glucose, amino acid, and lipid metabolism by releasing fly orthologs of insulin, glucagon, leptin, and other hormones [3]. Moreover, energy metabolism and the process of ATP production from nutrients are comparable between humans and *Drosophila* [4]. These similarities with the availability of powerful genetic tools and the suitability to perform large in vivo screenings make the fly a useful model system to study MetS.

Dynamical network biomarkers (DNB) theory has been developed to quantify the pre-disease state (or called “Mebyo”, meaning “not sick yet” in Japanese) or provide early-warning signals of the disease state during disease development [5,6]. The pre-disease state is an unstable state in which the healthy (homeostatic) state is changing into the disease state. This state is accompanied by early-warning signals, appearing just before the critical transition in various dynamical systems [7,8]. At this state, functional relationships between genes change dramatically, and gene expression levels fluctuate with strong correlations [9]. The DNB analysis has been used to identify the early signs of imminent disease or state transition including MetS, several cancers, Parkinson’s disease, embryonic stem cell differentiation, and so on [6,10,11,12,13,14,15].

Our previous study identified a group of collectively fluctuated genes with a significant correlation (namely DNB genes) in the adipose tissue of a mouse model of MetS, Tsumura Suzuki Obese Diabetes (TSOD) mice, at the pre-disease state [15]. Unexpectedly, these DNB genes are mostly associated with reproduction such as spermatogenesis and spermatid development [15]. We have demonstrated that fluctuations in the DNB gene expression at the pre-disease state are suppressed by a Kampo formula, a traditional Japanese medicine, Bofutsushosan extract, which is prescribed to T2D patients [16]. However, the potential role(s) for these DNB genes in the disease development of MetS has remained unknown.

To address this, we applied the DNB intervention analysis based on the stabilization method of control theory [17,18] to identify the potential therapeutic target from the list of DNB genes. We also took advantage of *Drosophila melanogaster* to perform a metabolic screening approach by starvation assay to investigate a role of candidate DNB genes from the DNB intervention analysis in development of MetS. Our metabolic screening identified Vasa (also known as DDX4), a DEAD-box RNA helicase [19], which is a conserved protein involved in the germline specification and gametogenesis in diverse organisms as a hit molecule. We confirmed its expression in the adipose tissue not only in a fruit fly but also in mammals in response to high-fat diet (HFD) or higher body mass index (BMI). Together, we show that Vasa could be a potential therapeutic target for preventing MetS.

## 2. Materials and Methods

### 2.1. The DNB Analysis and the DNB Intervention Analysis

DNB analysis is a method to find a pre-disease state, i.e., the state just before the critical transition from the healthy state to the disease state, where the fluctuation and correlation of mRNA expression levels of a group of genes increase due to the interaction among genes, based on the standard deviation of mRNA expression levels of each gene and the absolute value of their correlation coefficients [5,6,9]. As the next step after the detection of the pre-disease state, DNB intervention analysis has been developed to analyze which genes should be intervened in order to pull back from the pre-disease state to the healthy state without falling in a disease state [17,18]. This method is based on the stabilization method of control theory and extracts candidate genes for which intervention on gene expressions may be highly effective, by means of the eigenvector analysis of the sample covariance matrix created from the mRNA expression levels of genes in the pre-disease state. It should be noted that this method is used to narrow down candidate genes, and not all interventions on the selected candidates may be effective. It is also not possible to determine whether to suppress or promote them.

### 2.2. Fly Culture and Stocks

Flies were reared on a standard laboratory diet (8.5% cornmeal, 1.6% dry yeast, 7.5% glucose, 0.46% agar, 1% propionic acid mix, 1% Tegosept). Emerged adults were allowed to mate for 3–5 days in the bottle and transferred to the experimental diets. The normal diet (ND: 8.5% cornmeal, 5% yeast extract, 5% glucose, 0.46% agar, 1% propionic acid mix). To prepare the high-fat diet (HFD), 15% lard was added into the normal diet. For the *S_1_106-GS* driver, RU486 (Cayman Chemical, Ann Arbor, MI, USA) was dissolved in 95% ethanol and was used at a final concentration of 100 μM (the media is then referred to as ‘+RU486’). The control ND or HFD contained the same volume of 95% ethanol and is referred to as ‘−RU486’.

The fly stocks used in this study are as follows: *S_1_106-GS* (a gift from Dr. Pankaj Kapahi), *UAS-cys RNAi* (VDRC_104909), *UAS-cox8 RNAi* (VDRC_9108), *UAS-vasa RNAi* (BDSC_34950, VDRC_103427), *UAS-alpha Tub84B RNAi* (VDRC_33427), *UAS-cpa RNAi* (VDRC_100773), *UAS-aub RNAi* (VDRC_30124), *UAS-GFP RNAi* (VDRC_60103), *UAS-w RNAi* (BDSC_33644), *attP2* (BDSC_36303), *Canton-S* (a gift from Dr. Toshiro Aigaki), *w1118* (a gift from Dr. Pankaj Kapahi), *w*; *vasa::EGFP^KI^* (KYOTO_118616), and *UAS-EGFP* (BDSC_5431).

### 2.3. Starvation Assay

Emerged adults were allowed to mate for 3–5 days in the bottle and transferred to experimental diets (ND or HFD with/without RU486). Male flies (day 7) were transferred to vials containing 0.5% glucose and 0.5% agar. The flies were transferred to fresh vials every 24 h and deaths were recorded every 6–12 h.

### 2.4. Bulk RNA Sequencing (RNA-seq) Analysis

Total RNA was extracted from 15 male fat bodies (fly abdomen) of *S_1_106-GS > UAS-vas RNAi* flies using the TRIzol reagent (Thermo Fisher Scientific, Waltham, MA, USA) with the manufacture’s protocol. Extracted total RNA was treated with DNase (QIAGEN, Germantown, MD, USA) followed by further purification using the RNeasy Plus Mini Kit (QIAGEN). Three samples were prepared for each experimental group. Then, total RNA samples were forwarded to the Bioengineering Lab. Co., Ltd. (Kanagawa, Japan) for library preparation and RNA sequencing. An MGIEasy RNA Directional Library Prep Set (MGI Tech, Shenzhen, China) was used for library preparation. The library quality was verified using the Fragment Analyzer Systems with a dsDNA 915 Reagent Kit (Agilent Technologies, Santa Clara, CA, USA) and the Agilent 2100 Bioanalyzer with a High Sensitivity DNA Kit (Agilent Technologies). A single-stranded circular DNA library was prepared using an MGIEasy Circularization Kit (MGI Tech). The DNA nanoball (abbreviated as “DNB” only in this paragraph) was prepared using a DNB Rapid Make Reagent Kit (MGI Tech). RNA sequencing was performed using the DNBSEQ-T7 (MGI Tech). The paired-end 400 bp sequence data were analyzed as follows: The raw reads were filtered to remove the adaptors and low-quality bases using cutadapt (ver. 4.0) and sickle (ver. 1.33). Filtered reads were aligned to the *Drosophila* genome (BDGP6.46) using STAR (ver. 2.7.9a) and Samtools (ver. 1.17) was used to convert SAM format to BAM format. The read counts were calculated using featureCounts (ver. 2.0.3). Identification of differentially expressed genes and further downstream analysis were conducted with an integrative RNA-seq analysis platform, iDEP 2.01 [20] with the default parameters. RNA sequencing data have been deposited at the DDBJ under accession number PRJDB20100.

### 2.5. qRT-PCR

Total RNA was extracted from 12 male fat bodies (fly abdomen) or 5 male whole flies using the TRIzol reagent (Thermo Fisher Scientific) with the manufacture’s protocol. cDNA was synthesized using a PrimeScript RT reagent Kit with gDNA Eraser (TaKaRa Bio, San Jose, CA, USA). A total of 1 μg of total RNA was used per sample. The qPCR reaction was performed in triplicate on each of 8–9 independent biological replicates using GeneAce SYBR qPCR Mix α No ROX (NIPPON GENE, Tokyo, Japan). Error bars indicate SEM. Samples were normalized with an endogenous control, *Ribosomal protein L32* (*rp49/RpL32*). The primer sets used for this study are as follows:

*CG5966*-F: 5′-ACACCCTGGTGGACCTACC-3′, *CG5966*-R: 5′-AACAGCCATACACTCCGAAGC-3′, *bmm*-F: 5′-CAATAAGGGTCTGGCCAACTGGAT-3′, *bmm*-R: 5′-TAAGTCCTCCACCATTACTCTGGC-3′, *dHSL*-F: 5′-GCCTAAGGATCCATTCCTGTCG-3′, *dHSL*-R: 5′-CTCCATGGCTTCGTTGGATAAC-3′, *rp49*-F: 5′-CCACCAGTCGGATCGATATG-3′, and *rp49*-R: 5′-CACGTTGTGCACCAGGAACT-3′.

### 2.6. Immunohistochemistry

Flies were dissected in phosphate-buffered saline (PBS). Dissected fat bodies were fixed with 4% formaldehyde in PBS for 30 min. Samples were washed for 10 min three times with PBST (0.1% Triton X-100 with PBS). Blocking was performed with 5% goat serum in PBST for 2 h at room temperature. Samples were incubated with primary antibody overnight at 4 °C, were then washed for 10 min three times with PBST, and incubated with secondary antibody for 2 h at room temperature. Nuclei were stained using DAPI. Samples were mounted with VECTASHIELD hard set and analyzed by fluorescence microscope (KEYENCE, Osaka, Japan: BZ-X710). The following antibodies were used in this study: anti-rabbit GFP (Invitrogen, Waltham, MA, USA: 1/500), anti-mouse Vasa (NIG-FLY, Shizuoka, Japan: 1/50), anti-rabbit Alexa fluor 488 (Cell Signaling Technology, Danvers, MA, USA: 1/500), anti-mouse Alexa fluor 555 (Cell Signaling Technology: 1/500).

### 2.7. Lipid Staining

Flies were dissected in PBS. Dissected fat bodies were fixed with 4% formaldehyde in PBS for 30 min. Samples were washed for 10 min three times with PBS, then incubated with LipidTOX Deep Red (Thermo Fisher Scientific) working solution (1/500 in PBS) for 2 h at room temperature. Samples were mounted with VECTASHIELD with DAPI and analyzed by fluorescence microscope (KEYENCE: BZ-X710).

## 3. Results

### 3.1. The DNB Intervention Analysis Selects the Genes to Be Targeted for Therapeutics

**Figure 1:**
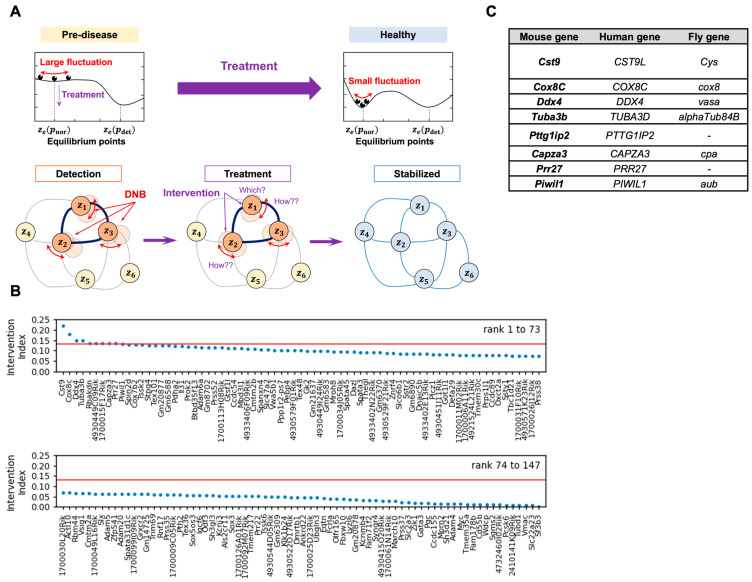
Selection of the candidate genes by the DNB intervention analysis. (**A**) A schematic diagram of the DNB intervention analysis. The DNB intervention analysis involves first detecting the pre-disease stage using high-dimensional low-sample-size (HDLSS) data. This is achieved by identifying a core set of genes (DNB members) with large fluctuations in correlation and variance, signaling an imminent transition to the disease state. High-dimensional statistical analysis is then applied to calculate the intervention index for each gene, ranking potential candidates for intervention based on their degree of fluctuations. The selected top-ranking genes were targeted for intervention, either through experimental or simulation-based methods, with the goal of transitioning the system from a high-fluctuation pre-disease state to a stable, healthy state. Successful intervention is validated by confirming reduced network fluctuations and sustained stability. (**B**) The result of the DNB intervention analysis. The intervention index expresses absolute values of elements of dominant eigenvector of sample covariance matrix of mRNA expression levels at pre-disease state. The blue circles indicate individual genes. The top 10 genes above the threshold (red line) were selected. (**C**) A list of eight candidate genes obtained from the DNB intervention analysis.

Spontaneous MetS model mice, TSOD mice, are an inbred strain that display MetS phenotypes that correspond to human [21]. Our previous study identified 147 DNB genes from the adipose tissue of TSOD mice at the pre-disease stage by DNB analysis [15]. To prioritize these genes for investigating their functions in the development of MetS, we applied DNB intervention analysis to find the candidate genes to be intervened (Figure 1A). Figure 1B shows the results obtained according to DNB intervention analysis, where the vertical axis implies the absolute values of each element of the dominant eigenvector of the sample covariance matrix with respect to mRNA expression levels at the pre-disease state obtained from the adipose tissue of TSOD mice. Higher absolute values in the vertical axis indicate higher intervention effectiveness. Accordingly, we selected top 10 genes with high values including *Cst9*, *Cox8c*, *Ddx4*, *Tuba3b*, *Rbakdn*, *4930449C09Rik*, *Pttg1ip2* (*1700015F17Rik*), *Capza3*, *Prr27*, and *Piwil1*. Then, we excluded lncRNA genes *Rbakdn* and *4930449C09Rik* to have eight candidate genes (Figure 1C).

### 3.2. Fat Body-Specific Knockdown of Vasa Abrogates the Effect of HFD in Fruit Fly

**Figure 2:**
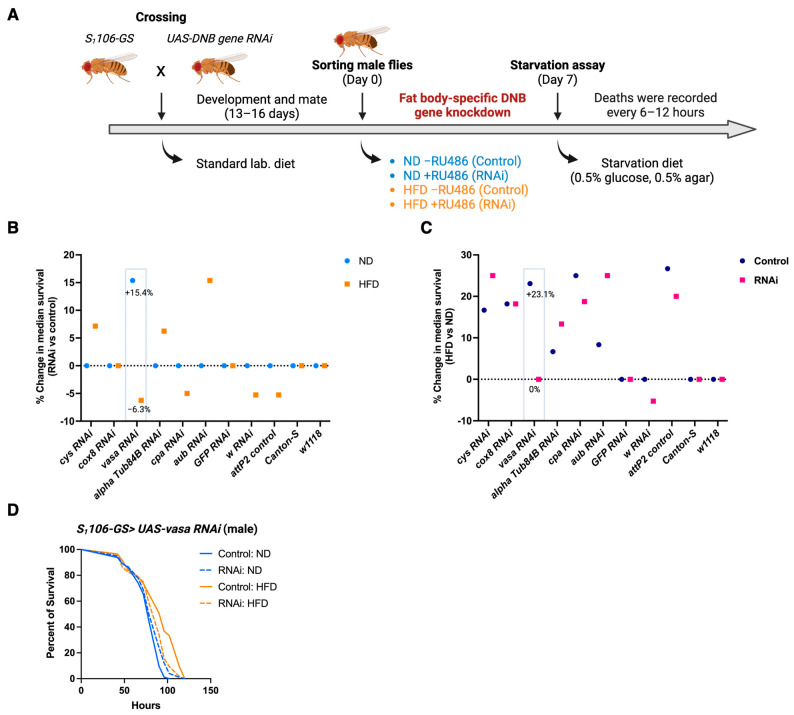
Fat body-specific *vasa* knockdown abrogates the effect of HFD on starvation resistance. (**A**) An experimental flow of the RNAi-mediated metabolic screening for DNB genes by starvation assay. (**B**) Percent changes in median survival both for ND (RNAi vs. Control) and HFD (RNAi vs. Control) are shown. (**C**) Percent changes in median survival both for Control flies (HFD vs. ND) and RNAi flies (HFD vs. ND) are shown. (**D**) Kaplan–Meier survival analysis of *S_1_106-GS > UAS-vasa RNAi* male flies are shown. Control: ND (*n* = 81) vs. Control: HFD (*n* = 57); *p* < 0.0001, Control: ND vs. RNAi: ND (*n* = 99); *p* = 0.0318, Control: HFD vs. RNAi: HFD (*n* = 64); *p* = 0.0043 by a Log-rank (Mantel–Cox) test. Representative survival curve of three independent assays.

To evaluate whether these genes are involved in metabolism, we conducted RNA interference (RNAi)-mediated knockdown screening for the fly ortholog of DNB candidate genes except *Pttg1ip2* and *Prr27* by observing their starvation resistance. Each DNB genes were knocked down in the fat body (equivalent to the adipose tissue and liver in mammals) using *S_1_106-GeneSwitch Gal4*, and the flies were fed either ND or HFD with/without RU486 before the starvation assay (Figure 2A). With the repeated experiments using two independent RNAi strains in each candidate, we found that fat body-specific *vasa* knockdown male flies consistently abrogated HFD-mediated increase in starvation resistance, while these flies enhanced starvation resistance upon ND compared to the control flies (Figure 2B–D). We confirmed similar results using another *vasa* RNAi strain (Supplementary Figure S1A). On the other hand, fat body-specific *vasa* knockdown female flies did not show a significant difference in starvation response (Supplementary Figure S1B). Together, these results suggest that Vasa play a role in fat body metabolism, especially in male flies.

### 3.3. Vasa Expressing Cells Are Increased in the Fat Body in Response to HFD in Fruit Fly

**Figure 3:**
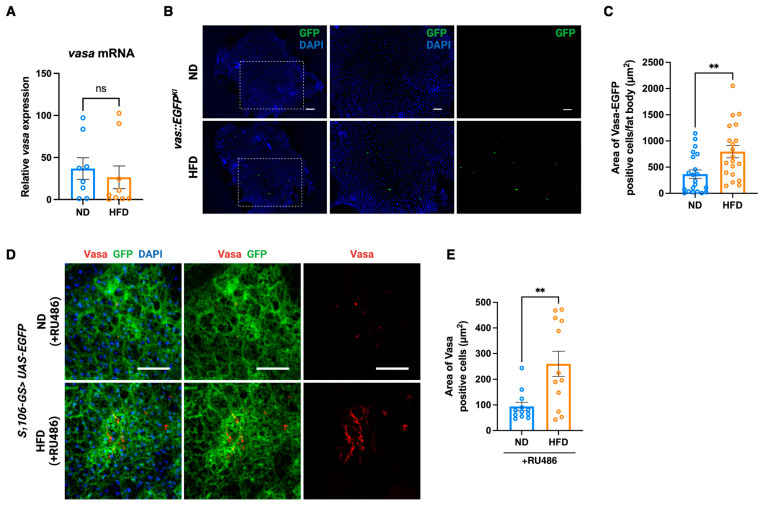
Vasa expressing cells are increased in the fat body in response to HFD. (**A**) *vasa* mRNA expression in dissected fat bodies from 3 days old male *w1118* flies. Error bars indicate SEM from eight (ND) or nine (HFD) independent biological replicates (ns: no significance by a two-tailed unpaired *t* test). (**B**) Immunostaining of Vasa-GFP in dissected fat bodies with anti-GFP antibody. *vas::EGFP^KI^* flies were fed ND (Top) and HFD (Bottom) for 3 days. Representative images (*n* = 20–21) are shown. Middle and right panels are magnified images of the white square in the left side panels. Scale bars indicate 100 μm (left) and 50 μm (middle and right). (**C**) Quantification of Vasa-GFP-positive cells from 20 to 21 of whole fat body images. Error bars indicate SEM (** *p* < 0.01 by a two-tailed unpaired *t* test). (**D**) Immunostaining of Vasa in dissected fat bodies with anti-Vasa antibody. *S_1_106-GS> UAS-EGFP* flies were fed ND (Top) and HFD (Bottom) for 3 days with RU486, respectively. Representative images (*n* = 12) are shown. Scale bars indicate 40 μm. (**E**) Quantification of Vasa positive cells from 12 of fat body images. Error bars indicate SEM (** *p* < 0.01 by a two-tailed unpaired *t* test).

Although Vasa is a well-known factor to be expressed and post-transcriptionally regulated in the germ cells [22,23,24], Vasa expression in the fat body has not been characterized. To investigate the expression level of Vasa in the fat body, we first searched *vasa* in the FLY CELL ATLAS (https://flycellatlas.org (accessed on 5 July 2024)), the cellular reference maps of the entire adult *Drosophila* based on the single-cell transcriptomes [25]. We confirmed that *vasa* mRNA is indeed expressed in several cells of the fat body. Next, we quantified *vasa* mRNA expression in the fat body under ND and HFD conditions. We found that *vasa* mRNA expression in the fat body was highly varied among samples, and we did not observe a significant difference in *vasa* mRNA expression between ND and HFD conditions at 3 days of age (Figure 3A). On the other hand, we found that Vasa expressing fat body cells were significantly increased in the HFD condition compared to ND on day 3, when we investigated Vasa expression using *vas::EGFP^KI^* flies [26] (Figure 3B,C). To confirm these results, we examined immunostaining for Vasa using the anti-Vasa monoclonal antibody [27]. We found similar results that Vasa expressing cells in the fat body from *S_1_106-GS > UAS-EGFP* flies increase upon HFD (Figure 3D,E). These results suggest that Vasa is post-transcriptionally regulated and plays a role in modulating the diet-dependent changes in fat body metabolism.

### 3.4. Fat Body-Specific Knockdown of Vasa Up-Regulates the Lipid Breakdown Pathways Upon HFD

**Figure 4:**
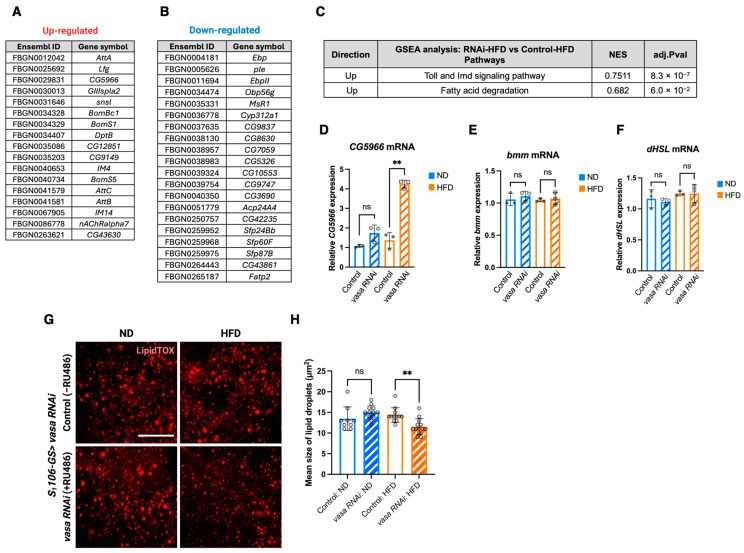
Fat body-specific *vasa* knockdown enhance upregulation of the fatty acid degradation pathway upon HFD during starvation. (**A,B**) Bulk RNA-seq analysis of dissected fat bodies from 7 days old male *S_1_106-GS > UAS-vasa RNAi* flies after 48 h starvation. The lists of up-regulated (**A**) and down-regulated (**B**) genes in the *vasa* RNAi flies compared with control flies upon HFD are shown. (**C**) GSEA pathway analysis of A. (**D**–**F**) *CG5966* (**D**), *bmm* (**E**), and *dHSL* (**F**) mRNA expressions in dissected fat bodies from 7 days old male *S_1_106-GS > UAS-vasa RNAi* flies after 48 h starvation. (**D**–**F**) Error bars indicate SD (ns: no significance, ** *p* < 0.01 by a two-tailed unpaired *t* test). (**G**) Lipid (LipidTOX) staining of dissected fat bodies from 7 days old male *S_1_106-GS > UAS-vasa RNAi* flies after 48 h starvation. Representative images (*n* = 9–12) are shown. Scale bar indicates 50 μm. (**H**) Quantification of lipid staining (**G**). (ns: no significance, ** *p* < 0.01 by a one-way ANOVA, Tukey’s multiple comparisons test).

To investigate the potential role of Vasa in fat body metabolism, we fed ND and HFD to fat body-specific *vasa* knockdown flies and control flies for 7 days followed by starvation for 48 h, which is the same conditions as our metabolic screening (Figure 2A), followed by bulk RNA-seq analysis using dissected fat bodies. Fat body-specific *vasa* knockdown resulted in the altered expression of 37 genes, with 17 being up-regulated and 20 down-regulated in the HFD condition (Figure 4A,B). GSEA pathway analysis using the KEGG pathway database revealed that up-regulated genes were associated with the Toll and Imd signaling pathway and the fatty acid degradation pathway (Figure 4C). On the other hand, down-regulated genes did not show any significant pathway from this analysis. We found that *CG5966*, a putative triacylglycerol lipase, which is an ortholog of pancreatic lipase-related protein 2 (PNLIPRP2) and PNLIPRP3, was up-regulated in the fat body-specific *vasa* knockdown flies upon HFD (Figure 4A). Thus, we verified these results by qRT-PCR and confirmed that *CG5966* was highly up-regulated in these flies in an HFD-dependent manner under starved conditions (Figure 4D). In *Drosophila* fat body, two major lipases, bmm (brummer) and dHsl (*Drosophila* hormone-sensitive lipase), have been shown to control triglyceride (TAG) levels redundantly [28]. However, fat body-specific *vasa* knockdown did not alter the expression of *Bmm* and *dHsl* both ND and HFD conditions (Figure 4E,F).

Next, we wanted to confirm that up-regulation of *CG5966* upon HFD in fat body-specific *vasa* knockdown flies resulted in changing their lipid storage. Fat body-specific *vasa* knockdown flies were starved for 48 h, and stored lipid in the fat body was stained using LipidTOX. Consistent with *CG5966* expression, we found that the size of the lipid droplets in the *vasa* knockdown flies upon HFD was significantly smaller than that in the control flies (Figure 4G,H). To gain mechanistic insights underlying the regulation of lipid metabolism by Vasa, we investigated 3′UTR of *CG5966* to search the Vasa binding motif using STREAM, a motif discovery algorithm [29]. We found that the (U)-rich motif, a known Vasa binding motif [30], in 3′UTR of *CG5966*, although the transcriptional regulatory mechanism of *CG5966* is still unclear (Supplementary Materials Figure S2A). We next conducted a protein network analysis of Vasa using STRING, a database of known and predicted protein–protein interactions [31]. STRING provided the results that Vasa interacts with the subunits of mitochondrial ATP synthase (Supplementary Figure S2B,C), suggesting its involvement in energy metabolism, yet it did not show the Vasa-*CG5966* interaction. Together, these results suggest that Vasa negatively regulates *CG5966* expression, and that may be involved in HFD-induced obesity.

### 3.5. DDX4/Ddx4 Expressions Are Altered in the Adipose Tissue of Mouse and Human

**Figure 5:**
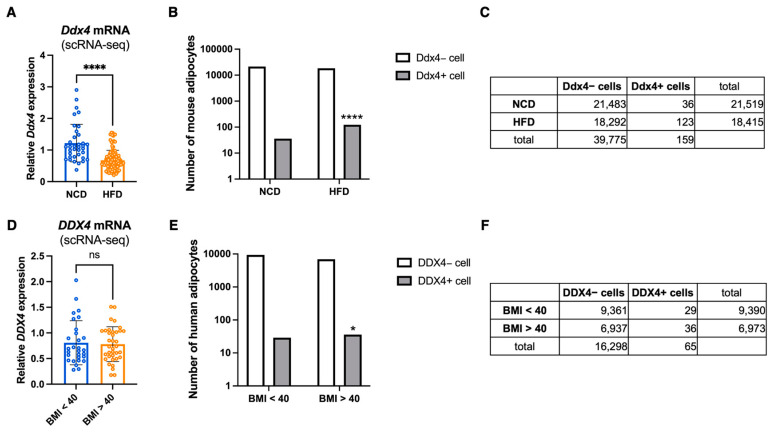
*DDX4/Ddx4* expression are altered in the adipose tissues of HFD-fed mice and the patients with high body mass index. (**A**–**F**) scRNA-seq data extracted from a single-cell atlas of human and mouse white adipose tissue of the Single Cell PORTAL (https://singlecell.broadinstitute.org/single_cell (accessed on 13 September 2024)). (**A**) *Ddx4* mRNA expression in mouse adipocytes. Error bars indicate SD (**** *p* < 0.001 by a two-tailed unpaired *t* test). (**B**,**C**) Number of *Ddx4* expressing cells and non-*Ddx4* expressing cells calculated from a dataset of A (**** *p* < 0.001 by a Chi-square test). (**D**) *DDX4* mRNA expression in human adipocytes. Error bars indicate SD (ns: no significance by a two-tailed unpaired *t* test). (**E**,**F**) Number of *DDX4* expressing cells and non-*DDX4* expressing cells calculated from a dataset of D (* *p* < 0.05 by a Chi-square test).

Although our metabolic screening was performed using fruit flies, our original DNB gene candidates were selected from the mammalian model, TSOD mice. This prompted us to search whether Vasa has a potential translational value for human patients with a high BMI. For that purpose, we used the Single Cell PORTAL (https://singlecell.broadinstitute.org/single_cell (accessed on 13 September 2024)) database, containing 50,676,975 cells of 764 single-cell RNA-seq studies, to explore the *Ddx4/DDX4* expression in mouse and human adipose tissues. From this database, we utilized a dataset of a single-cell atlas of human and mouse white adipose tissue (WAT) [32]. As shown in Figure 5, we first compared the expression levels of *Ddx4* in mouse adipocytes between normal chow diet (NCD) and HFD-fed mice. We found that the expression level of *Ddx4* showed a slight but significant decrease in the HFD condition (Figure 5A). However, we noticed that the number *Ddx4* expressing adipocytes were strikingly increased upon HFD (Figure 5B,C). We next observed the expression levels of *DDX4* in human adipocyte from obese (BMI > 40) or non-obese (BMI < 40) patients. Due to the characteristics of the recruited patients, the threshold of BMI was decided as 40 [32]. We found that the number of *DDX4* expressing adipocytes was significantly increased in the patients with higher BMI (Figure 5E,F), which is consistent with *Ddx4* expression in mice upon HFD, while we did not observe any difference in the *DDX4* expression levels between obese and non-obese patients (Figure 5D). These results suggest that DDX4 expression in the adipose tissue is associated with obesity. These observations support our notion that Vasa/DDX4 is a potential therapeutic target for preventing MetS.

## 4. Discussion

Recent advances in the field of basic medical research using transcriptomics including microarray, bulk RNA-seq, and scRNA-seq technologies with associated computational tools achieved to detect early signs and predict the onset of metabolic diseases [33,34] as well as accelerate drug discovery [35]. However, biological validation of mathematical prediction methods is still insufficient. Herein, we demonstrated for the first time that data-driven mathematical approaches using the DNB analysis and the DNB intervention analysis with an in vivo research model such as *Drosophila melanogaster* enable us to identify the novel therapeutic target genes for MetS at a preclinical stage. Our findings suggest that the DNB intervention analysis is effective and highly promising for applications to other diseases to identify genes, which may be a contributing factor in disease development.

Using the DNB intervention analysis, a control theory-based mathematical modeling, and a metabolic screening approach using a simple model organism, *Drosophila melanogaster*, we identified Vasa/DDX4 as a novel regulator of lipid metabolism. We found that fat body-specific *vasa* knockdown up-regulates lipid breakdown pathways upon HFD resulting in abrogates HFD-induced enhancement of starvation resistance. We also showed that Vasa/DDX4 expressing cells in the adipose tissue were increased upon HFD or obesity conditions not only in fruit flies but also in mammals.

The HFD-induced obesity model has been established in *Drosophila* and displays elevation of TAG levels, activation of immune response, heart dysfunction, and shortened lifespan [36,37,38]. TAG storage provides glycerol and fatty acids (FAs) by lipolysis as a major energy source in situations of energy depletion or increased energy demand [39]. Therefore, TAG levels in fruit flies are often correlated with their starvation resistance. For example, both HFD-fed flies and fat body-specific *Brummer*, an ortholog of the mammalian *ATGL* (adipose triglyceride lipase), knockdown flies increased TAG levels resulted in enhanced starvation resistance [40,41]. Consistent with these observations, our starvation screening demonstrated that flies in most of the genotypes showed enhancement of starvation resistance upon HFD compared with ND. This confirmed that our HFD-induced obesity model has been established. However, flies in some genotypes did not show a similar trend, suggesting that the genetic background may affect the HFD-induced enhancement of starvation resistance.

Vasa was first identified in *Drosophila* as a translational regulator in the germ line [42,43]. Over the past decades, Vasa has been shown to have broad functions including translational regulation of specific mRNAs and Piwi-interacting RNA (piRNA) generation, which contribute to cell fate determination within the germ line [30,44,45,46]. Importantly, recent studies have revealed that Vasa functions in somatic cells including embryonic cells, regenerative tissues, and tumorigenic cells of diverse organisms [47]. Our data demonstrate that Vasa/DDX4 may function in the adipose tissue to contribute to the development of HFD-induced obesity. DDX helicases are a conserved group of RNA-binding proteins that regulate various cellular RNA processes and physiology [48,49]. It is noteworthy that several DDX helicases including DDX1, DDX5, and DDX17 are involved in maintaining lipid homeostasis [50,51,52]. It has been shown that DDX17 is elevated and transcriptionally represses *Cyp2c29* gene expression by cooperating with CCCTC binding factor (CTCF) and DDX5 in the murine model of metabolic dysfunction associated steatohepatitis (MASH) induced by HFD [52].

Our findings provide evidence that Vasa/DDX4 plays a role in lipid metabolism by regulating *CG5966*, a triglyceride lipase, expression. Although the motif analysis demonstrated the potential interaction between Vasa and *CG5966* mRNA, Vasa/DDX4-associated mRNAs in the adipose tissue remain unclear. However, our finding in the interaction between Vasa and mitochondrial ATP synthases by STRING suggests that Vasa may be involved in regulating energy homeostasis by balancing fatty acid beta-oxidation and ATP synthesis. Mitochondria preferentially uses glucose for ATP production, but lipids provide an alternative fuel source when glucose availability is low, such as during starvation [53]. In *Drosophila*, overexpression of fatty acid beta-oxidation genes, *fabp* (*fatty acid binding protein*) and *Dci* (*Dodecenoyl-CoA delta-isomerase*), has been shown to enhance their starvation resistance as well as extends lifespan [54]. Our observations that fat body-specific *vasa* knockdown flies abrogated HFD-mediated increase in starvation resistance in males but not in females suggest Vasa may contribute to the mitochondrial energy production pathway in the sex- and diet-dependent manner.

Our study demonstrates the existence of sex differences in the effect of fat body-specific *vasa* knockdown on starvation resistance as only male flies showed a significant response. Many previous studies have demonstrated that Vasa/DDX4 knockdown phenotypes differ between different organisms, or between sexes of the same species including *Drosophila*, *C. elegans*, zebrafish, Medaka (*Oryzias latipes*), and mice [55,56,57,58,59]. For example, *vasa* mutant flies display sterility in females but not in males [55], whereas in mice it causes sterility in males but not in females [59]. Moreover, in *Drosophila*, *vasa* is expressed in somatic cells of the embryonic gonad in a male-biased manner, and is regulated post-transcriptionally [60]. The mechanism of these variable Vasa/DDX4 functions among different species and sexes is largely unknown, yet our findings suggest that Vasa/DDX4 could play an important role in the regulation of lipid metabolism in a sex-dependent manner. However, it should be acknowledged that this study has limitations, and caution should be used when interpreting our sex-dependent effect of *vasa* knockdown. We used the RU486-inducible *S_1_106-GeneSwitch Gal4* to knockdown *vasa* in the fat body. RU486 has been shown to regulate the lifespan and metabolism in female *Drosophila* in a mating-dependent manner [61]. Thus, knocking down effects of *vasa* in female flies may be masked by RU486-inducible metabolic shift. Further studies are needed to fully understand the mechanisms by which Vasa/DDX4 regulates lipid metabolism.

Other than the DNB intervention analysis, several structural network controllability and perturbation analyses have been shown to understand and influence gene expression networks. Vinayagam et al. [62] demonstrated that the structural controllability of the human protein interaction network could identify disease genes and drug targets by classifying proteins as indispensable, neutral, or dispensable based on their impact on network controllability. Devkota and Wuchty [63] further highlighted the role of control proteins in regulating molecular pathways, showing that a small set of proteins governs numerous pathways and serves as potential drug targets. Additionally, Markowetz [64] reviewed methods for leveraging gene perturbation screens through network analysis, offering insights into how perturbation effects can be mapped onto cellular networks to identify therapeutic interventions. These studies underscore the potential of network-based methods for advancing therapeutic strategies and predictive modeling of gene expression.

Together, our findings open exciting avenues for future research to understand the role of Vasa/DDX4 in the soma as well as to improve our understanding of the molecular mechanisms underlying the sexual dimorphic regulation of lipid metabolism.

## Data Availability

RNA sequencing data in this study have been deposited at the DDBJ (https://www.ddbj.nig.ac.jp) under accession number PRJDB20100.

## Supplementary Materials

The following supporting information can be downloaded at: https://www.mdpi.com/article/10.3390/cells14060415/s1, Figure S1: Fat body-specific *vasa* knockdown abrogates the effect of HFD on starvation resistance in males but not in females. Figure S2: The potential interacting targets of Vasa.
