# Supplementary material for: Integration of Dynamical Network Biomarkers, Control Theory and Drosophila Model Identifies Vasa/DDX4 as the Potential Therapeutic Targets for Metabolic Syndrome
Source: Cells. 2025 Mar 12;14(6):415. doi: 10.3390/cells14060415 (PMC11941168; doi:10.3390/cells14060415)
Supplement: Supplementary file 1 [file cells-14-00415-s001.zip › cells-3470046-supplementary.pdf]

Supplementary Figure S1.

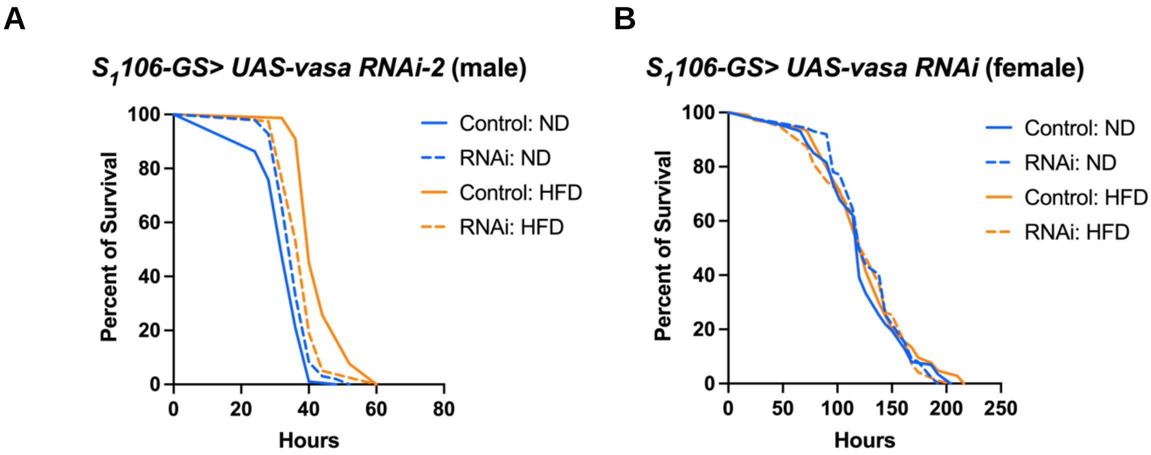

Supplementary Figure S1. Fat body-specific *vasa* knockdown abrogates the effect of HFD on starvation resistance in males but not in females.
(A) Kaplan–Meier survival analysis of *$S_1$106-GS > UAS-vasa RNAi* (VDRC_103427) male flies are shown. Control: ND (n=95) vs Control: HFD (n=78); $p < 0.0001$, Control: ND vs RNAi: ND (n=97); p=0.0013, Control: HFD vs RNAi: HFD (n=79); $p < 0.0001$ by a Log-rank (Mantel–Cox) test. Representative survival curve of three independent assays. **(B)** Kaplan–Meier survival analysis of *$S_1$106-GS > UAS-vasa RNAi* (BDSC_34950) female flies are shown. Control: ND (n=87) vs Control: HFD (n=104); p=0.3403, Control: ND vs RNAi: ND (n=87); p=0.6148, Control: HFD vs RNAi: HFD (n=95); p=0.5602 by a Log-rank (Mantel–Cox) test. Representative survival curve of three independent assays.

Supplementary Figure S2

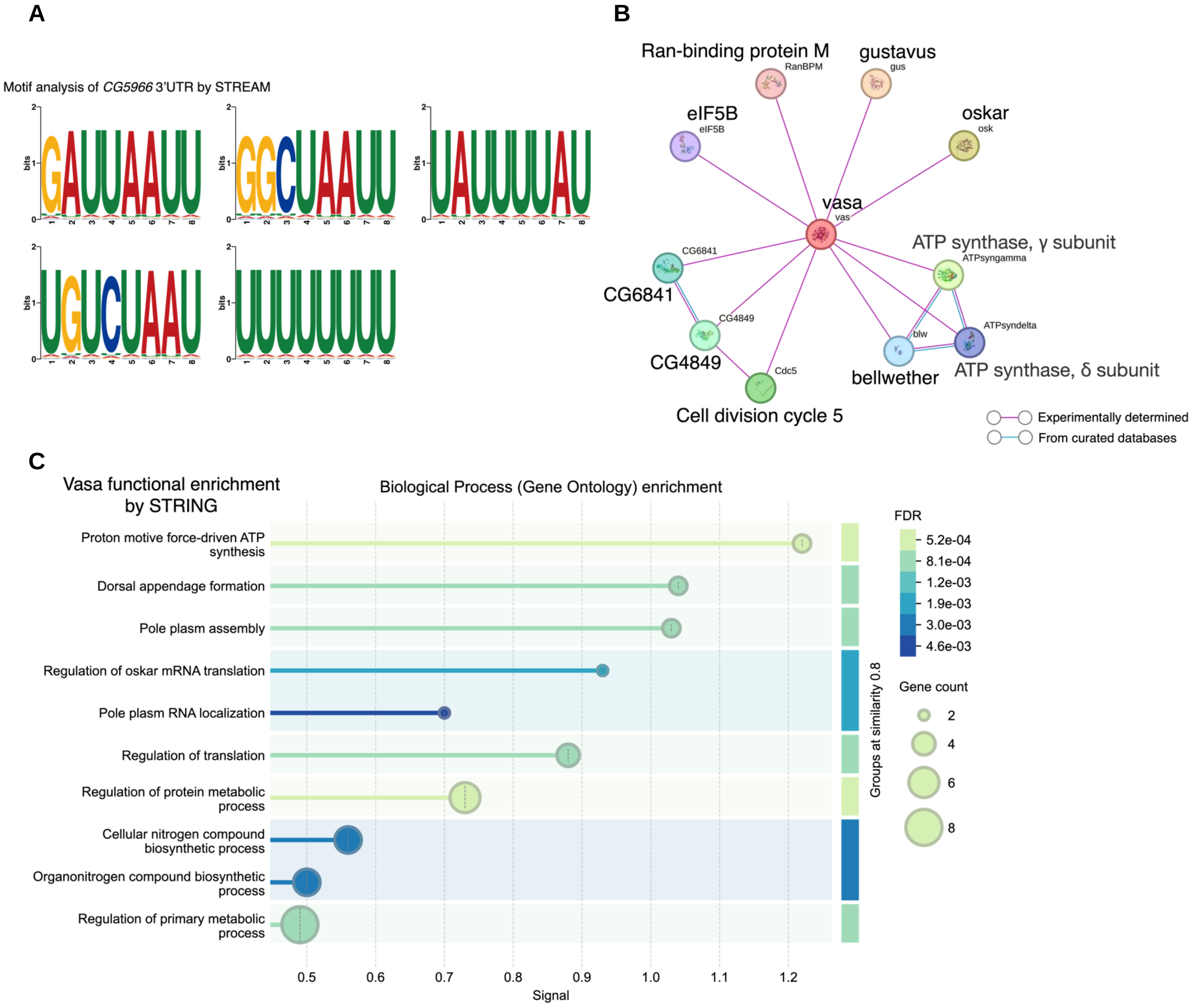

Supplementary Figure S2. The potential interacting targets of Vasa.
(A) The motif analysis of *CG5966* 3'UTR by STREAM. The five identified motifs are shown. **(B** and **C)** The protein network analysis of Vasa by STRING. (B) The predicted functional partners of Vasa are shown. (C) The result of functional enrichment visualization with a default parameter setting is shown.
